# DH-GarlicNet: a precise identification method for garlic damage based on the improved residual network

**DOI:** 10.3389/fpls.2026.1801346

**Published:** 2026-03-19

**Authors:** Zhuang He, Xiaodan Ma, Yiyang Jiang, Gang Chen, Yun Gao, Xin Wang, Tao Qi, Zhennan Xia

**Affiliations:** School of Information Engineering, Changchun College of Electronic Technology, Changchun, China

**Keywords:** deep learning, garlic detection, image classification, image processing, residual network

## Abstract

With the increasing level of agricultural automation, the detection of damaged garlic has become an important task to ensure food quality and improve production efficiency. This paper presents a novel damaged garlic detection model -DH-GarlicNet. This model is based on the ResNet34 architecture and innovatively integrates depthwise convolution, SE attention module, and SiLU activation function, aiming to significantly improve the accuracy of damage detection. With the introduction of depthwise convolution, the model can efficiently extract subtle features in garlic images; the SE module enhances the focus on critical regions; and the SiLU activation function optimizes the representation of nonlinear features. The experimental results show that the DH-GarlicNet model achieves 98.95% detection accuracy and 0.0793 loss in the damaged garlic detection task, which improves the accuracy by 7.37% and reduces the loss by 0.2324 with respect to the original model, and significantly outperforms the traditional detection algorithm. The Grad-CAM visual analysis demonstrates the model’s ability to focus on the damaged regions, proving its advantages in target recognition and local feature learning. This study provides an accurate solution for the automated detection of damaged garlic, which has a strong application prospect.

## 1 Introduction

Garlic, as an important agricultural crop (Parreño et al., 2023), not only occupies an important position in the global food culture but also has rich nutritional value and medicinal properties (Gorinstein et al., 2007). However, due to its fragile nature, garlic is susceptible to external damage or bacterial contamination during production, storage, transportation, and marketing, thus affecting its quality, safety, and market value (Bhatwalkar et al., 2021). Damaged garlic may not only reduce its nutrient content but also become a breeding ground for germs, thus affecting consumers’ food safety (Chowdhury et al., 2023). Therefore, ensuring that garlic remains intact at all stages and being able to accurately and efficiently detect whether it is damaged or not is a key part of ensuring food safety and improving the quality of agricultural products.

At present, the traditional method of garlic damage detection mainly relies on manual inspection and mechanical equipment (Yang et al., 2022a). Manual inspection is usually through the naked eye to observe the appearance and morphological changes of garlic to determine whether it is damaged or not. Although this method is simple and intuitive, due to the complex shape of garlic and the existence of multiple forms of damage, manual inspection is difficult to ensure efficiency and accuracy (Hussamadin et al., 2023). In addition, manual operation is easily affected by environmental lighting, staff experience, and other factors (Connelly et al., 2022), resulting in instability and poor repeatability of the results, and the speed of manual inspection is also difficult to meet the needs of large-scale production (Tian et al., 2022). Mechanical inspection methods mainly rely on some simple sensors (Hassani and Dackermann, 2023), such as pressure sensors, temperature sensors, or visual sensors, to determine whether the garlic is damaged or not by detecting its hardness, temperature, or surface characteristics. Some scholars have also used electronic noses to detect varieties of garlic (Trirongjitmoah et al., 2015). However, these traditional sensor technologies tend to exhibit low sensitivity and accuracy in the face of complex and variable damage types, and often fail to accurately recognize subtle damage, especially when the appearance and texture of garlic change very subtly (Mardanshahi et al., 2025). Therefore, although traditional detection methods have helped to some extent, they often fail to meet the requirements for high precision and efficiency in modern agricultural production.

With the rapid development of artificial intelligence technology, especially the great breakthroughs made by deep learning in the field of computer vision, the method of agricultural product damage detection based on image recognition has gradually become a hot spot of research (Saleem et al., 2021). Deep learning technology can automatically extract features from images and learn complex patterns by training on a large amount of image data, so as to realize the automatic recognition and classification of agricultural damage (Nyakuri et al., 2024; Song et al., 2025). Compared with traditional rule-based algorithms, deep learning has stronger expressive ability and adaptability, and can recognize more multi-dimensional and complex damage features (Li et al., 2022). Among them, the convolutional neural network, as a classical deep learning architecture, has achieved remarkable results in image classification and target recognition tasks (Abdullah and Ahmed, 2023; Ghimire et al., 2022; He and Xiao, 2024; Hu et al., 2018; Khan et al., 2020; Liu et al., 2017). CNN, through multi-layer convolution and pooling operations, can efficiently extract local features in images and synthesize global information, and has strong automatic feature learning and expression ability (Chen et al., 2025). In the field of damage detection of agricultural products, deep learning methods, especially CNN-based image recognition technology, are able to accurately determine whether garlic is damaged or not by automatically analyzing its appearance image, and even further identify the type and degree of damage.

Pham Thi Quynh Anh and other scholars introduced a modified multi-class model and a multi-label model that utilized CNN to classify two labels of a garlic bulb after root trimming. The first label includes good, bad, untrimmed, and scratched classes, and the second label consists of clean and muddy classes. The modified multi-class model achieved a classification accuracy of 82.9% while the multi-label model gave a better classification performance of minor classes, with an overall accuracy of 95.2%. With the addition of a background image class, classification accuracies of both multi-class model and multi-label model increased to 91.8% and 98.0%, respectively (Anh et al., 2022). Jinhwan Ryu and other scholars proposed a method based on a multispectral fast camera to detect heat stress in southern-type garlic, and it was found that the LS-SVM model performed optimally in this task (Ryu et al., 2023). Helong Yu and other scholars proposed a deep learning method based on Residual Network (ResNet) and FasterNet to efficiently identify the origin and variety of grain. The method solves the deep network gradient vanishing problem by combining the residual connection structure of ResNet (Yu et al., 2024), and at the same time, improves the computational efficiency of the model by utilizing the lightweight design of FasterNet (Yu et al., 2025). Ke Yang and other scholars developed a deep learning-based system for automated garlic root cutting, achieving a 96% success rate in experimental trials (Yang et al., 2022b). Lizhi Fang and other scholars proposed a capacitive sensing-based method for garlic clove orientation detection, achieving 96.75% accuracy using deep learning models (Fang et al., 2024). Currently, there are relatively few research studies on the detection of damage to garlic, and a systematic detection method has not yet been developed. In a previous study, object detection algorithms such as YOLO were employed to detect damaged garlic (Gao et al., 2026). A drawback of this approach is the significant time required for initial annotation of the garlic. Although garlic, as an important economic crop, is crucial for its post-harvest treatment and quality assurance, existing research has mostly focused on garlic yield prediction, disease identification, or planting optimization, and there is limited exploration of intelligent detection technologies for specific damage situations, such as mechanical damage, transportation, and storage damage.

Therefore, addressing the demand for damage detection in garlic during actual agricultural production, this study proposes an intelligent detection method based on deep learning. One of the primary challenges currently facing the garlic industry in the post-harvest processing stage is the lack of efficient and accurate damage detection methods. To overcome this technical bottleneck, this study innovatively adopts a two-stage technical framework involving segmentation followed by classification.

## 2 Materials and methods

### 2.1 Data acquisition and pre-processing

**Figure 1:**
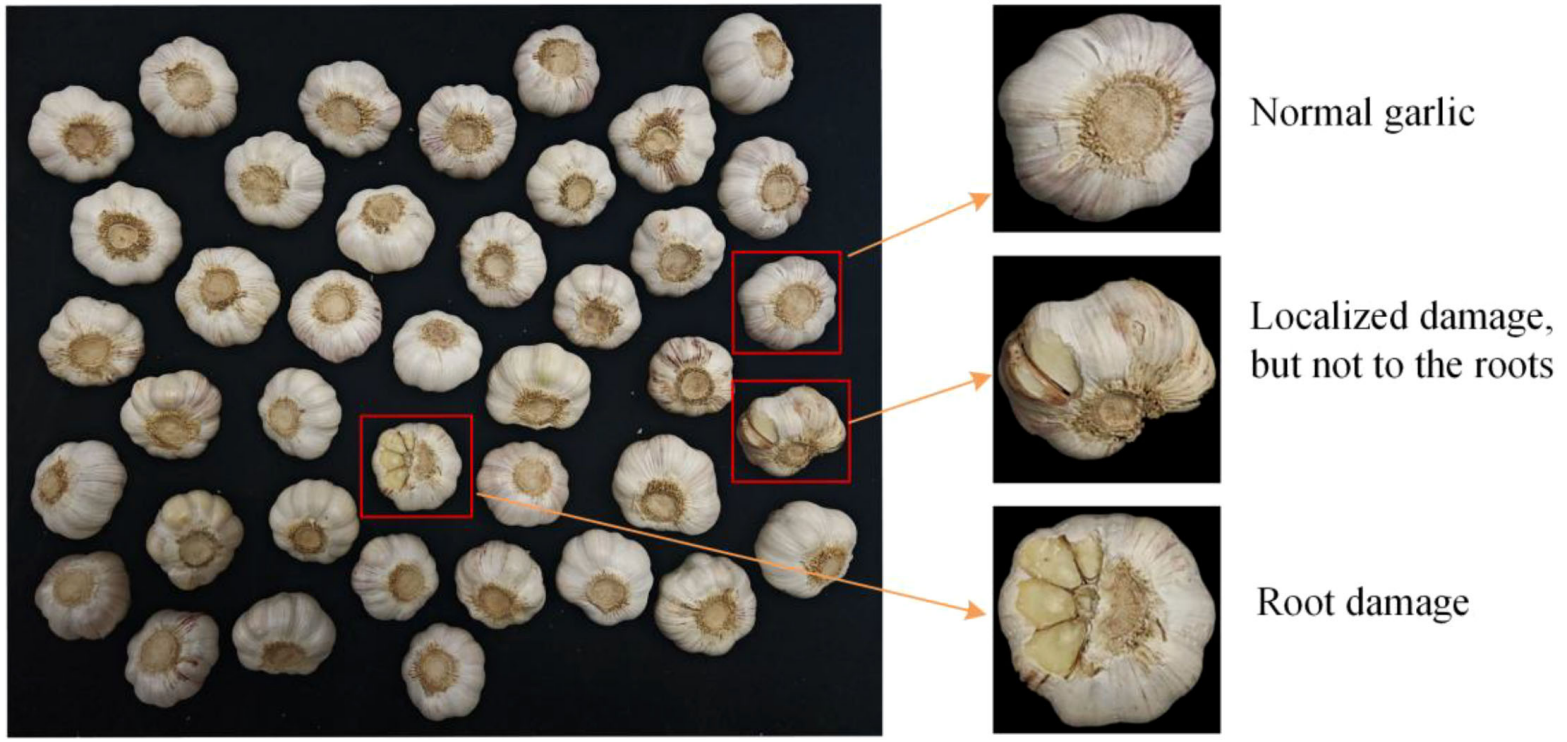
Garlic dataset presentation.

This study utilizes a garlic dataset, as presented in **Figure 1** (Gao et al., 2026), comprising samples cultivated in Heze City, Shandong Province, China. Reflecting practical production requirements, the dataset categorizes garlic into three distinct conditions: intact, locally damaged, and root-damaged. To simulate real-world handling and storage scenarios—where garlic is typically transported on conveyor belts—images were captured against a black cloth background. This setup enhances the realism and applicability of the data, aiming to provide reliable support for quality classification, damage detection, and related research. All images were acquired using an Honor Magic 6 device.

Intact garlic: Characterized by a complete and uniform appearance, firm and plump cloves, along with smooth, unwrinkled skin. Such garlic demonstrates high quality and marketability.

Locally damaged garlic (root intact): Exhibits superficial imperfections such as minor abrasions, scratches, or slight clove deformation, while the internal structure and root remain unaffected. These specimens retain edibility and general market value despite cosmetic flaws.

Root-damaged garlic: Shows visible breakage or decay at the root area. This type of damage compromises water and nutrient uptake, diminishes storage stability, and renders the garlic unsuitable for both planting and prolonged sale.

**Figure 2:**
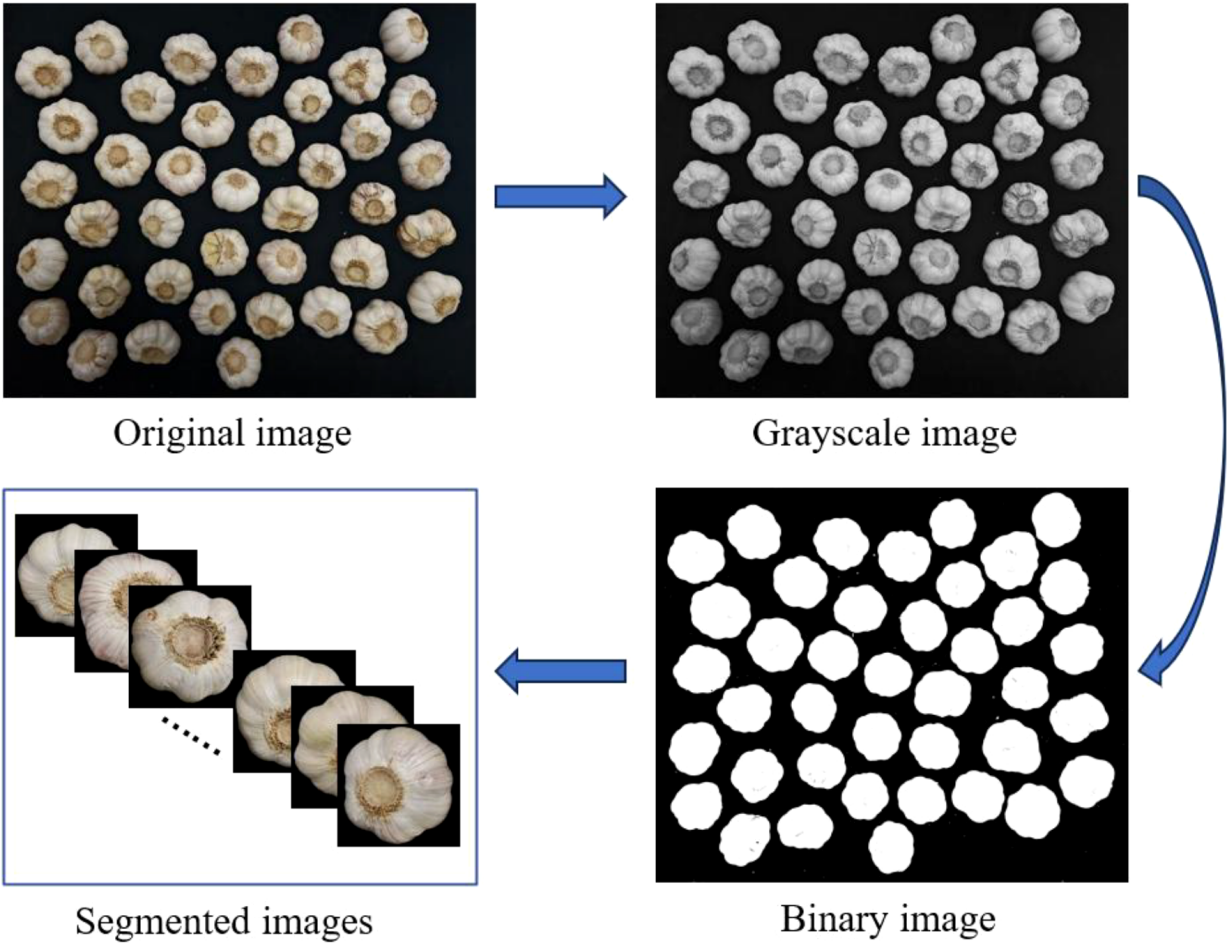
Image segmentation process.

In our previous research, we employed object detection methods to identify garlic (Gao et al., 2026). In this study, we propose a novel approach for garlic damage detection: utilizing a combination of image processing and image classification techniques. The image segmentation process, illustrated in **Figure 2**, uses a threshold-based method. First, a grayscale version of the original image is created. Then, a threshold value is chosen to categorize the image’s pixel values into two groups: the target region and the background. For computational simplicity and efficiency, this study first analyzed the grayscale histograms of a representative image subset during threshold determination. This subset exhibited a bimodal distribution with a distinct trough near 0.3, effectively separating garlic regions from the background. Therefore, the threshold was set at 0.3, where pixel values below 0.3 are set to 0 (black) and values above 0.3 are set to 1 (white), resulting in a binarized image. Next, the binarized image is multiplied pixel-by-pixel with the original image. The pixels with a value of 1 in the binarized image (representing the target) retain their values from the original image, while pixels with a value of 0 (representing the background) are set to black, removing the background. Finally, a contour extraction algorithm is applied to the processed image, extracting the edges of the garlic and isolating the target region. This process effectively separates the object from the background, enabling further analysis.

In this experiment, the captured raw garlic images contain some low-quality samples that need to be eliminated, such as blurred images due to shooting jitter, corrupted files during storage and transmission, and invalid frames with serious background interference or incomplete targets. If these low-quality data are used directly for model training without strict screening, they will negatively affect the performance of deep learning algorithms in many ways. From the perspective of model optimization, low-quality training data can significantly increase the variance of the optimization process, making it difficult for the loss function to converge stably. In more serious cases, when the percentage of invalid data is too high, the model may even learn pseudo-features that are completely irrelevant to the target task (Koziarski and Cyganek, 2018). Therefore, after removing useless images in this experiment, a total of 487 images were obtained. Data augmentation was performed using online random noise injection and random rotation. Finally, the dataset was divided into training, validation, and test sets in a 6:2:2 ratio. Detailed data specifications are shown in **Table 1**.

**Table 1:** Details of the data set used in this study.

Label	Data type	Original image	Remove useless image	Data enhancement	Training set	Validation set	Test set
1	Locally damaged garlic	102	95	285	171	57	57
2	Root-damaged garlic	98	96	288	173	57	58
3	Normal garlic	304	296	888	533	178	177

### 2.2 Model building

**Figure 3:**
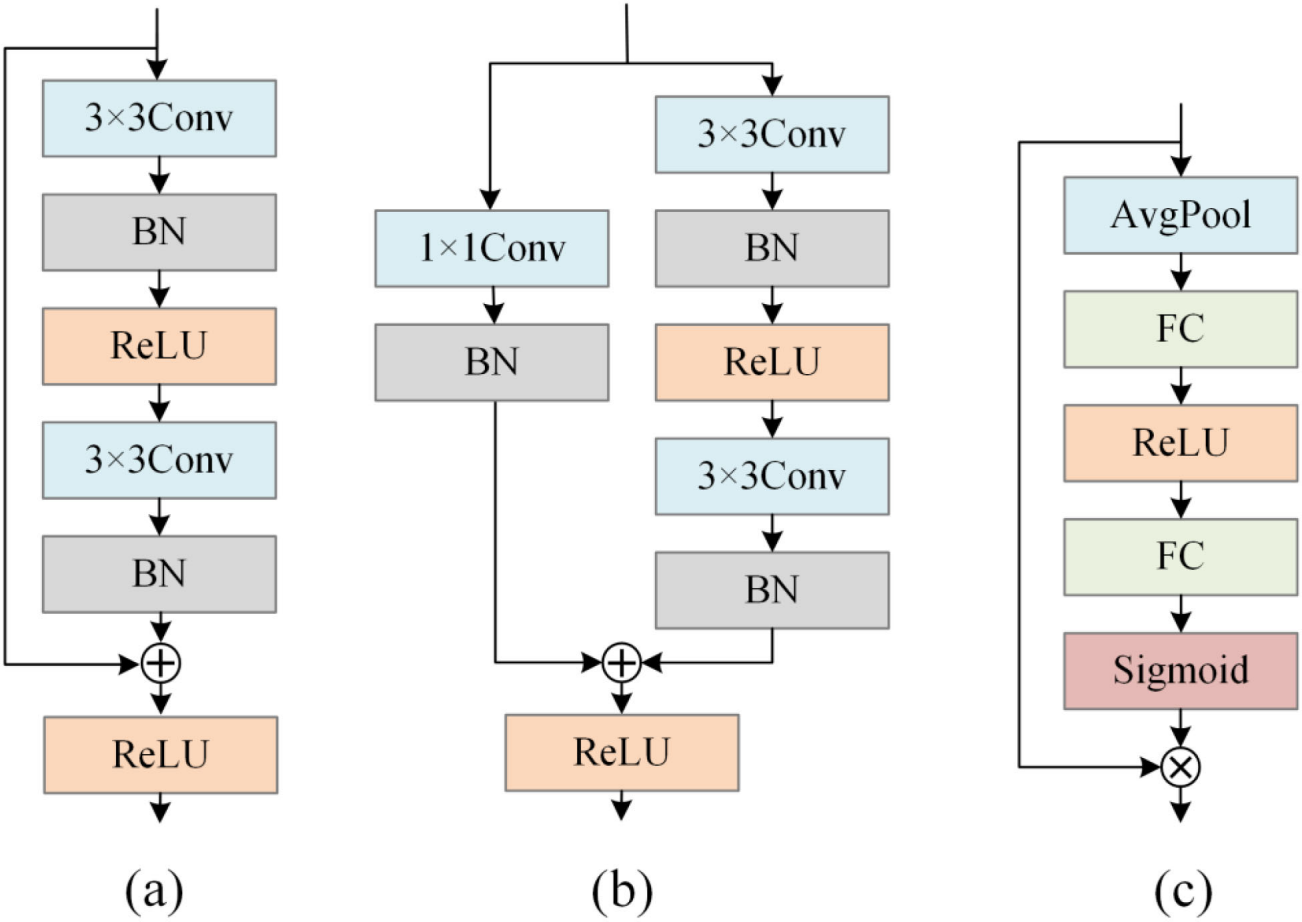
The modules used in this study are: **(a)** residual structure, **(b)** downsampling structure, and **(c)** SE attention mechanism.

As shown in **Figure 3**, this study adopts the residual connections, downsampling structures, and SE attention mechanism from ResNet34 as the basic framework (He et al., 2015). The residual connections in ResNet34 construct an efficient gradient propagation path through cross-layer identity mapping. This design not only alleviates the problem of gradient disappearance in deep network training but also enables the network to better learn multi-level feature representations. In the garlic damage detection task, damage features may simultaneously contain local minor damages and overall morphological changes. The residual structure allows the network’s layers to flexibly reuse the underlying features, avoiding the problem of feature information attenuation layer by layer in traditional network structures, which is particularly important for accurately identifying different degrees and types of damage.

The core of ResNet34 lies in constructing identity mappings through residual connections, enabling the network to learn residual functions rather than direct mappings. For a residual block, let the input be x and the output be y. Its mathematical expression is shown in Equation 1.

(1) $$\begin{array}{c} y=F\left(\text{x},\left\{Wi\right\}\right)+x \end{array}$$

Here, F(x,{Wi}) denotes the residual mapping to be learned, typically composed of two stacked 3×3 convolutional layers, each followed by a batch normalization layer. The second convolution omits ReLU activation, with the outputs summed before activation. When input and output dimensions or spatial dimensions mismatch, shortcuts require introducing a 1×1 convolution for projection adjustment. Its mathematical expression is shown in Equation 2.

(2) $$\begin{array}{c} y=F\left(\text{x},\left\{Wi\right\}\right)+W_{s}x \end{array}$$

$W_{s}$ denotes a 1×1 convolution kernel, with a stride matching that of the downsampling layers in the residual branches. The entire ResNet34 network commences with an initial 7×7 convolution layer (stride 2) followed by a 3×3 max-pooling layer, downsampling the input image from 224×224×3 to 56×56×64. Subsequently, the network progresses through four stages, each containing multiple residual blocks as described above: The first stage comprises three identity mapping blocks (64 channels). The second stage first employs a downsampling projection block to increase channels to 128 and reduce dimensions to 28×28, followed by three identity blocks. The third stage first uses a projection block to increase channels to 256 and reduce dimensions to 14×14, followed by five identity blocks. The fourth stage first uses a projection block to increase the channels to 512 and reduce the size to 7×7, followed by two identity blocks. Finally, the network outputs classification results through global average pooling and a fully connected layer. This residual structure enables effective gradient propagation as the network deepens, thereby supporting training at greater depths.

The design of the downsampling structure fully considers the multi-scale characteristics of images. By using a phased approach of stride convolution and skip connections for downsampling, the network can gradually build a feature pyramid from local details to global semantics. In garlic damage detection, surface scratches, mold, etc., are small-scale damages that require high-resolution features, while overall rot and other large-scale changes require a wider perception field. This downsampling strategy retains important feature information through 1×1 convolution in the skip connections during the process of spatial dimension reduction, avoiding the problem of loss of minor damage features caused by simple pooling operations, and ensuring that the network has good detection capabilities for different scales of damage.

The introduction of the SE attention mechanism is to cope with the complex interference in actual detection scenarios. During garlic image acquisition, there may be factors such as uneven illumination, surface reflection, or soil adhesion. The SE module implements adaptive calibration of feature channels through the channel attention mechanism, enabling the network to autonomously strengthen the feature channels related to damage and suppress irrelevant background interference. This dynamic feature adjustment capability is particularly suitable for handling sample images of varying quality in actual acquisition environments. This architecture, which combines residual connections, multi-scale downsampling, and attention mechanisms, not only inherits the advantages of classic models but also forms an efficient solution for the damage detection task of agricultural products through the collaborative effect of each component.

**Figure 4:**
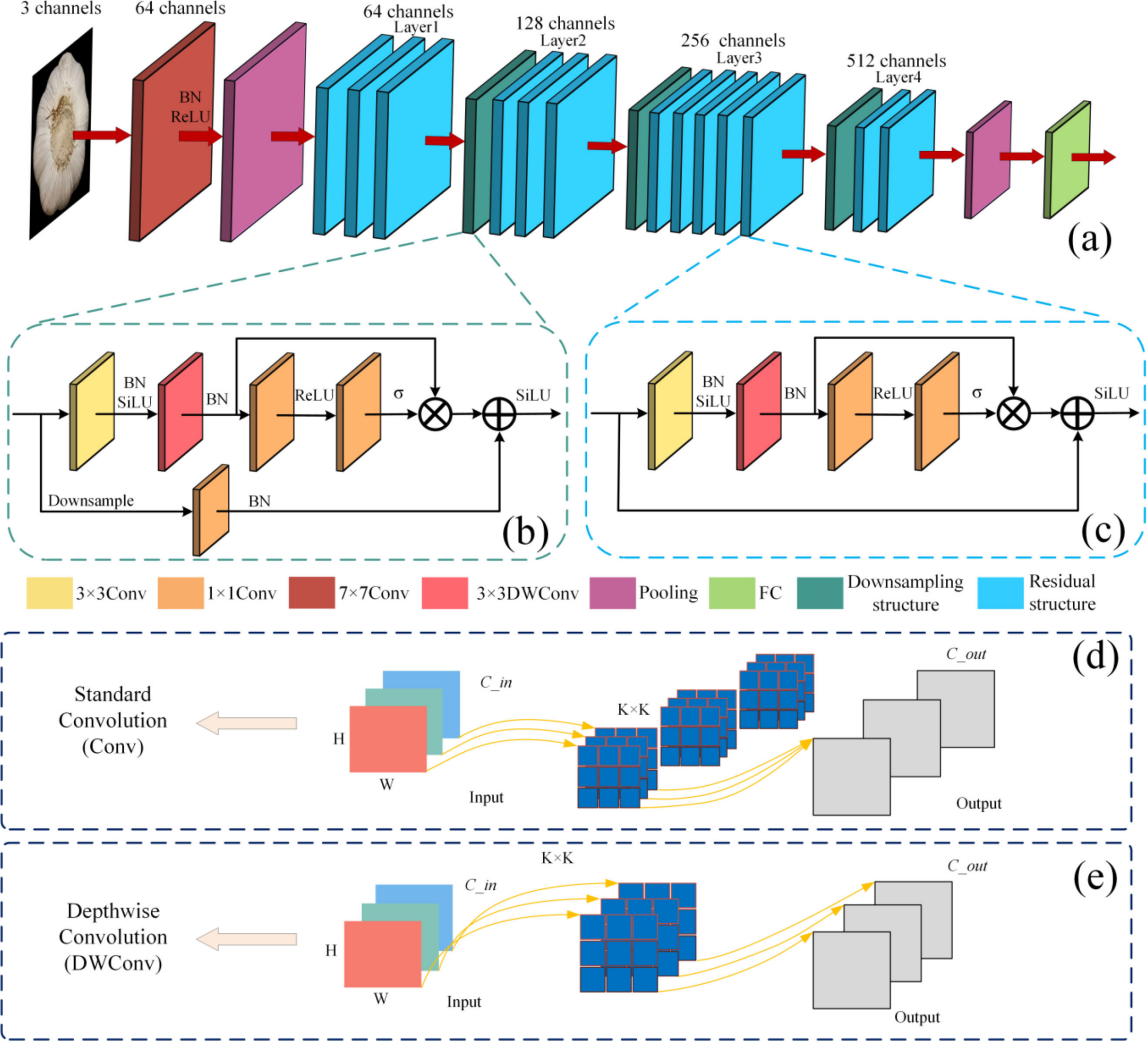
Schematic of the model structure, **(a)** shows the backbone architecture of DH-GarlicNet, **(b)** shows the schematic of the downsampling structure, **(c)** shows the schematic of the residual structure, **(d)** shows the schematic of the structure of the Standard Convolution (Conv), and **(e)** shows the schematic of the structure of the Depthwise Convolution (DWConv).

As shown in **Figure 4a**, this study proposes a deep learning algorithm called DH-GarlicNet, tailored to the specific characteristics of garlic. The model is built upon ResNet34 (He et al., 2015), which achieves a critical balance in deep neural network design by offering strong feature extraction capabilities while maintaining high computational efficiency. The 34-layer depth enables the network to capture complex features across multiple levels, from low-level textures to high-level semantic patterns, making it particularly well-suited for extracting damage-related features in garlic, such as localized bruising or root damage.

As shown in **Figures 4b, c**, DH-GarlicNet adopts a two-layer residual structure. On the basis of the traditional residual structure, it adds a secondary residual branch, creating a more complex feature fusion method. This nested design functions similarly to the SE attention mechanism, both aiming to adaptively calibrate features, though they follow different implementation approaches. The nested residual connection preserves the benefits of the original residual network in reducing gradient vanishing by establishing a multi-layer feature interaction channel, while further enhancing the network’s ability to adjust feature channels dynamically. The inner residuals concentrate on extracting local features, while the outer residuals handle the integration and calibration of global features. Notably, it enhances the detection of small-scale damage. The nested structure fosters complementary feature representations at different levels, and this multi-level feature fusion is a key factor in improving DH-GarlicNet’s performance.

In this research, a 3 × 3 Depthwise Convolution (DWConv) is employed instead of the traditional 3 × 3 Standard Convolution (Conv). This approach separates the processes of channel mixing and spatial feature extraction in standard convolution. It encourages the network to focus on learning the most important spatial patterns within each channel by preserving the topology of the input channels through independent, channel-by-channel spatial convolutions. As illustrated in **Figures 4d, e**, DWConv promotes better feature learning by isolating channels spatially, preventing early mixing of channel information as seen in Conv, and allowing the network to maintain a cleaner representation of spatial features. This characteristic is particularly useful when dealing with subtle damage in garlic images. DWConv operates on each input channel independently, whereas Conv convolves the entire input feature map. The number of convolution kernels in DWConv matches the number of input channels, with each kernel processing a single channel, while Conv typically has more kernels than input channels, with each kernel handling multiple channels. Let C_in and C_out denote the input and output channel numbers, respectively, K^2 represent the size of the convolution kernel, and the image size is H×W.

The number of parameters in Conv is as shown in Equation 3:

(3) $$\begin{array}{c} K^{2}\times C_{in}\times C_{out} \end{array}$$

The computational load of Conv is as shown in Equation 4:

(4) $$\begin{array}{c} K^{2}\times C_{in}\times H\times W\times C_{out} \end{array}$$

The number of parameters of DWConv is as shown in Equation 5:

(5) $$\begin{array}{c} K^{2}\times \frac{C_{in}}{G_{in}}\times C_{out} \end{array}$$

The computational load of DWConv is as shown in Equation 6:

(6) $$\begin{array}{c} K^{2}\times \frac{C_{in}}{C_{in}}\times H\times W\times C_{out} \end{array}$$

Meanwhile, this study introduces the SiLU activation function, which softly weights the original inputs by a Sigmoid function, and SiLU realizes the feature selection ability similar to the attention mechanism. This dynamic adjustment property enables the network to adaptively strengthen important features and suppress noise. The mathematical expression of the SiLU function is shown in Equation 7:

(7) $$\begin{array}{c} SiLU\left(x\right)=x\cdot \sigma \left(x\right) \end{array}$$

Where x is the input value and σ denotes the sigmoid function, the mathematical expression of the sigmoid function is shown in Equation 8.

(8) $$\begin{array}{c} \sigma \left(x\right)=\;\frac{1}{1+e^{-x}} \end{array}$$

Notably, SiLU(x) ≈ x when x tends to +∞; SiLU(x) tends to 0 when x tends to -∞; SiLU(0) = 0.5 when x is equal to 0; and the whole function is smooth and everywhere differentiable. The activation function combines the properties of linear rectification (ReLU) and sigmoid gating, which retains the sparse activation advantage of ReLU and realizes a more delicate feature regulation capability through sigmoid weighting.

**Figure 5:**
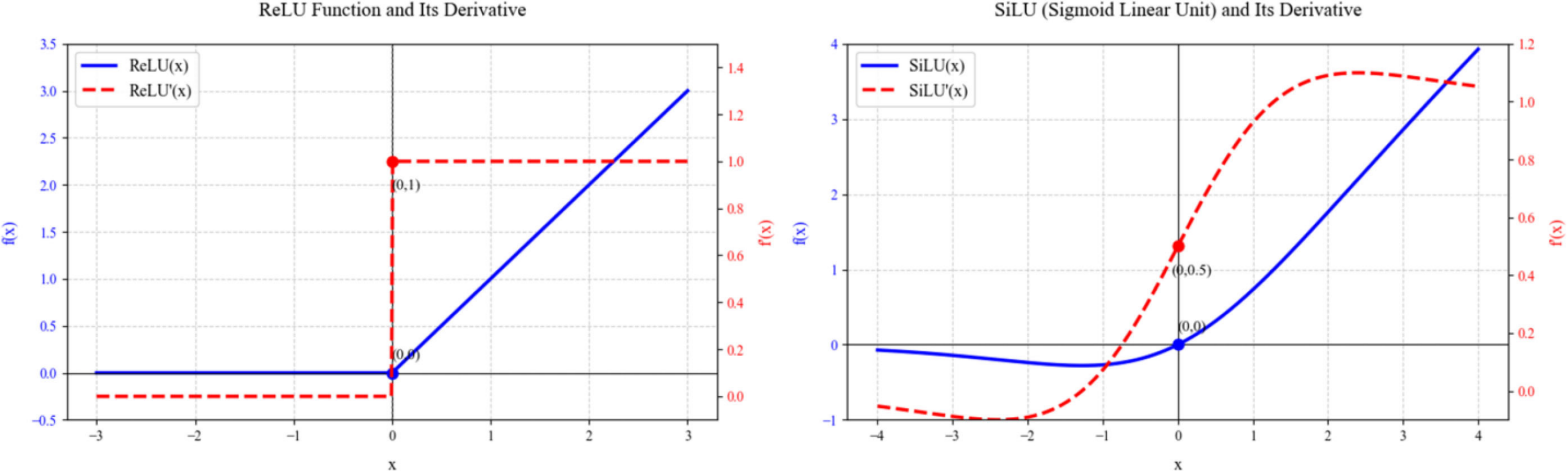
The curves and derivative curves of the ReLU activation function and the SiLU activation function.

As shown in **Figure 5**, the ReLU function maintains linear growth when the input is greater than zero, and outputs zero when the input is less than zero. This hard truncation characteristic can effectively introduce nonlinearity and alleviate the problem of gradient vanishing, but it also leads to the “Dead ReLU” phenomenon, where some neurons may never be activated. Additionally, ReLU is not differentiable at zero, and its derivative undergoes a sudden change from 0 to 1 at this point, which can affect the stability of the optimization process to some extent. In contrast, SiLU achieves a smooth nonlinear transition by multiplying the input by the sigmoid function. Its output retains a small but non-zero response in the negative range and approximately linear growth in the positive range. This design makes SiLU differentiable everywhere and has a continuous and smooth change in its derivative, which retains the advantages of ReLU in promoting sparse activation while avoiding the problem of neuron death.

This study chose SiLU mainly based on its comprehensive advantages in deep learning tasks. Firstly, the smooth characteristic of SiLU makes the model training process more stable, with more coherent gradient flow, which helps alleviate the problems of gradient vanishing or explosion. Secondly, the non-zero response in the negative range of SiLU retains more information flow, enhancing the model’s expressive power, which is particularly important for learning complex features. Moreover, the self-gating mechanism of SiLU (the product of the input and the sigmoid function) enables it to adaptively adjust the activation intensity of neurons, a dynamic nonlinearity that is more flexible than the fixed threshold of ReLU. Although the computational cost of SiLU is slightly higher than that of ReLU, the performance improvement it brings is usually sufficient to offset this cost.

### 2.3 Experimental environment and parameter settings

This study details the training parameters for the proposed network model. The input image size was fixed at 224×224 pixels, with a batch size of 8 and a base learning rate of 0.01. The model was trained for 30 epochs using Stochastic Gradient Descent (SGD) as the optimizer. All experiments were conducted on a workstation equipped with an Intel Xeon Gold 6246R CPU (3.4 GHz) and an NVIDIA Quadro RTX 8000 GPU (48GB VRAM), running Windows 10. The software environment included Anaconda3 (2021.11), PyCharm as the compiler, and PyTorch 1.2.1 built on Python 3.8.3. To ensure consistency, all algorithms were executed under identical hardware and software configurations.

### 2.4 Evaluation indicators for the model

In supervised learning, confusion matrices serve as a fundamental tool for evaluating classification model performance. The matrix organizes predictions against ground truth labels: columns correspond to predicted classes, while rows represent actual classes. For a binary classification task, the matrix consists of four key components. True Positives (TP): Cases where both the actual and predicted labels are positive, False Positives (FP): Negative instances incorrectly predicted as positive, False Negatives (FN): Positive instances misclassified as negative, True Negatives (TN): Correctly identified negative cases. The structure of a binary confusion matrix is illustrated in **Table 2**.

**Table 2:** Confusion matrix of a binary classification problem.

Confusion matrix		Actual results	
		Positive	Negative
Forecast Results	Positive	TP	FP
	Negative	FN	TN

Accuracy (Acc), Precision (P), Recall (R), and F1-score (F1) are derived from the confusion matrix and serve as key metrics for evaluating the classification performance of a model. The corresponding formulas and brief descriptions of these metrics are provided in **Table 3**.

**Table 3:** Formulas and brief descriptions of each evaluation indicator.

Evaluation indicators	Formulas	Brief description
Accuracy (Acc)	$Acc=\;\frac{TP+TN}{TP+FP+FN+TN}$	The ratio of the number of correctly predicted positive and negative samples to the total number of samples.
Precision(P)	$P=\;\frac{TP}{TP+FP}$	The ratio of the number of correctly predicted positive samples to the total number of samples predicted to be positive.
Recall(R)	$R=\;\frac{TP}{TP+FN}$	The ratio of the number of correctly identified positive samples to the total number of actual positive samples.
F1-score(F1)	$F1=2\times \;\frac{\text{Precision}\times \text{Recall}}{\text{Precision}+\text{Recall}}$	The reconciled mean of precision and recall.

## 3 Results and discussion

### 3.1 Results of the ablation experiment

This study validated the effectiveness of each key component in DH-GarlicNet through systematic ablation experiments, using ResNet34 as the base architecture with only standard convolution and ReLU activation functions. The model achieved 91.58% accuracy on the test set, highlighting the need for improved feature extraction in the base model. After substituting Conv with DWConv, the accuracy increased to 96.84%, demonstrating the advantage of deep convolution in preserving spatially detailed features. Incorporating the SE module on top of DWConv further boosted the accuracy to 97.89%. The final model, with the SiLU activation function, achieved 98.95% accuracy, which is a 7.37 percentage point improvement over the base model. The performance enhancements clearly illustrate a synergistic effect: DWConv aids in feature detail retention, the SE module enables intelligent feature selection, and SiLU optimizes the nonlinear feature representation. This combination led to the highest classification performance. Details are provided in **Table 4**.

**Table 4:** Partial results of ablation experiments.

DWConv	SE block	SiLU	Acc(%)	P(%)	R(%)	F1(%)	Loss
			91.58	87.10	90.13	88.50	0.3117
✓			96.84	97.07	94.20	95.50	0.1635
	✓		95.11	95.76	95.56	95.62	0.2231
		✓	94.33	94.35	94.12	94.24	0.2576
✓	✓		97.89	97.50	96.17	96.80	0.1130
	✓	✓	97.76	97.45	96.45	97.12	0.1102
**√**	**√**	**√**	98.95	98.23	98.03	98.10	0.0793

As shown in the comparative data in **Table 5**, replacing the second 3×3 Conv layer in the ResNet34 residual block with a 3×3 DWConv yields significant advantages across all key lightweight metrics, fully demonstrating the effectiveness of this structural improvement in enhancing model efficiency. In terms of parameter count, the modified model reduces from 21.8 M to 10.5 M, representing a decrease exceeding 50%. This change primarily stems from the batching mechanism of deep convolutions, which makes the layer’s parameter count linearly proportional to the number of input channels rather than quadratically. This significantly reduces the overall parameter size of the model. Correspondingly, the model’s weight size also shrinks from 83.3 MB to 42.1 MB, offering significant benefits for storage-constrained deployment scenarios. In terms of computational load, the model’s requirement decreased from 3.66 GFLOPs to 1.82 GFLOPs, also achieving a 50% reduction. This decrease in computational complexity directly translates to improvements in latency and throughput: inference latency decreased from 5.15 ms to 4.09 ms, while processing speed increased from 152 FPS to 201 FPS, representing performance gains of approximately 20% and 32%, respectively. Notably, the latency improvement slightly underperformed the theoretical computational reduction. This discrepancy may stem from operator implementation efficiencies in deep convolutions on actual hardware, where parallel optimization for DWConv remains less mature than for standard convolutions on certain hardware platforms. Overall, this structural refinement achieves dual benefits of model lightweighting and inference acceleration by replacing a single convolutional layer while preserving the core architecture of ResNet34. It significantly reduces the number of parameters, memory footprint, and computational overhead, while simultaneously delivering a notable increase in throughput performance.

**Table 5:** The comparison of the lightweight effectiveness of DWConv.

Convolution type	Parameters(M)	GFLOPs	Delay(ms)	Weight(MB)	FPS
ResNet34-Conv	21.8	3.66	5.15	83.3	152
ResNet34-DWConv	10.5	1.82	4.09	42.1	201

As shown in **Table 6**, the DH-GarlicNet model proposed in this study demonstrates outstanding performance and high stability in the five-fold cross-validation results for the garlic bulb recognition task. Overall evaluation reveals that the model achieved recognition accuracy exceeding 98.5% across all five validation folds, with a peak accuracy of 98.95% and an average accuracy of 98.67%. This demonstrates the model’s ability to effectively learn and recognize key features within the garlic bulb dataset, while exhibiting strong robustness to different training data partitions. In balancing precision and recall, the model also performed outstandingly, with F1 scores consistently ranging between 98.10% and 98.57%, averaging 98.43%. This fully demonstrates that the model maintains high precision while achieving good recall during recognition, resulting in balanced and reliable overall performance. Regarding loss function values, all folds maintained extremely low losses around 0.08, with the lowest reaching 0.0786. This not only confirms the model’s high prediction confidence but also indicates well-converged training without overfitting. Analyzing fold-specific performance: Fold 4 achieved the lowest loss and an F1 score of 98.55%, representing the optimal overall validation result. Although the fifth fold achieved the highest accuracy, its recall and F1 score were relatively lower, suggesting potential misclassification of a very small number of samples in this fold. However, the overall fluctuation was minimal. In summary, the DH-GarlicNet model demonstrates outstanding recognition accuracy and stable generalization capabilities in the garlic bulb identification task. The experimental results fully validate the reliability and effectiveness of this model in practical application scenarios.

**Table 6:** The five-fold cross-validation results of the DH-GarlicNet model.

Time	Acc(%)	P(%)	R(%)	F1(%)	Loss
Fold-1	98.51	98.85	98.23	98.50	0.0817
Fold-2	98.83	98.44	98.23	98.57	0.0825
Fold-3	98.52	98.54	98.56	98.43	0.0811
Fold-4	98.56	98.64	98.45	98.55	0.0786
Fold-5	98.95	98.23	98.03	98.10	0.0793

### 3.2 Comparative results before and after model improvement

**Figure 6:**
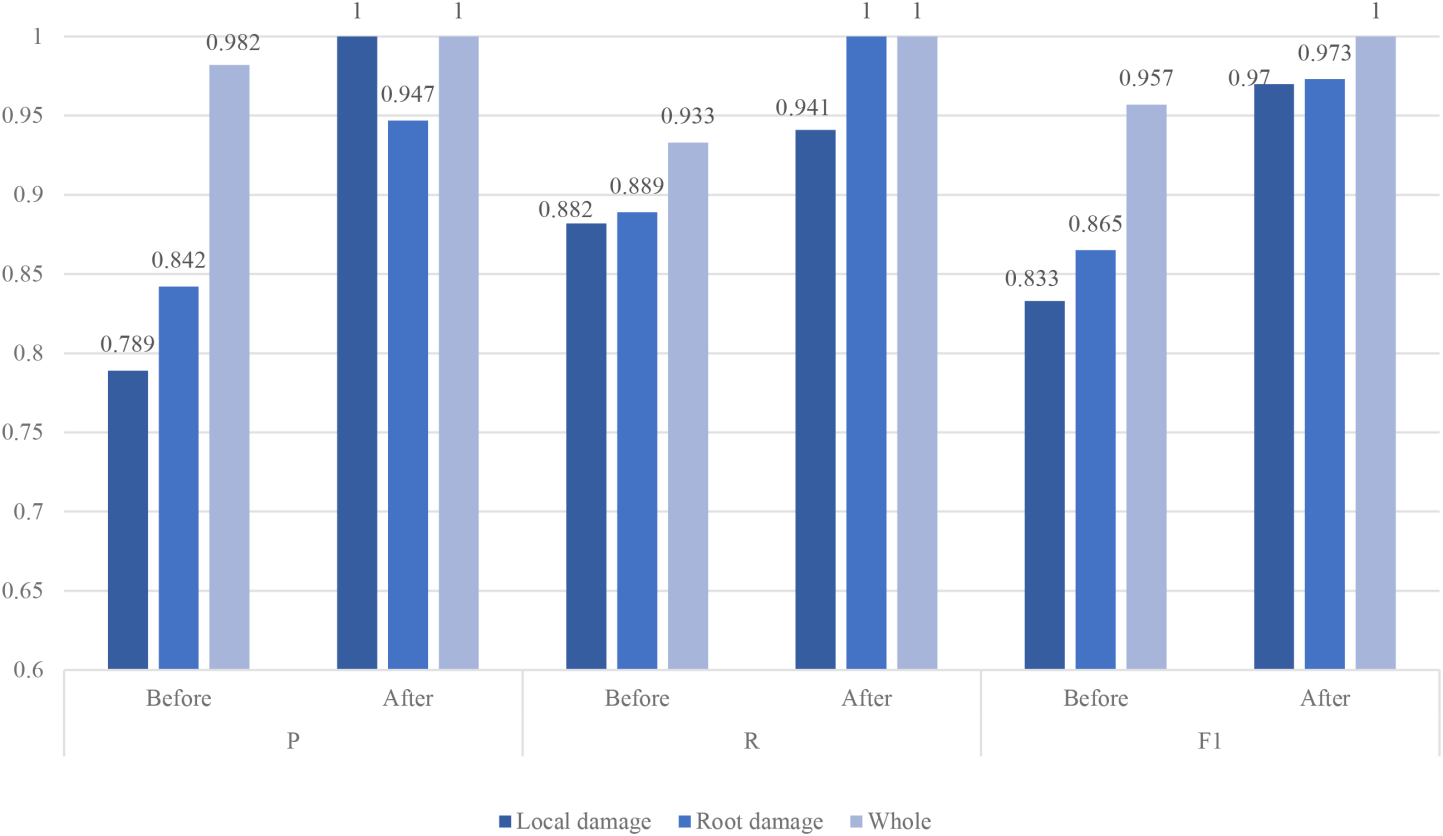
Comparison of each category before and after model improvement.

In this study, the enhanced recognition performance of the model across different categories is validated through comprehensive comparison experiments. As shown in **Figure 6**, the improved model outperforms the original on all evaluation metrics, particularly excelling in the recognition of key categories. Notably, the model achieves perfect performance on intact garlic, with precision, recall, and F1 score all reaching 1, indicating its ability to accurately identify healthy garlic samples. For root-damaged garlic, the model attains a recall of 1, successfully detecting all root-damaged samples without any missed detections. When identifying locally damaged garlic, the model also reaches a precision of 1, ensuring all identified samples are genuinely locally damaged, completely eliminating misdiagnoses. Besides these individual successes, the model shows significant overall improvement across all metrics compared to the original, enhancing the recognition balance for each category and eliminating the weaknesses found in some categories in the original model. These advancements together contribute to a substantial boost in the model’s overall performance, making it more effective for detecting actual damaged garlic.

**Figure 7:**
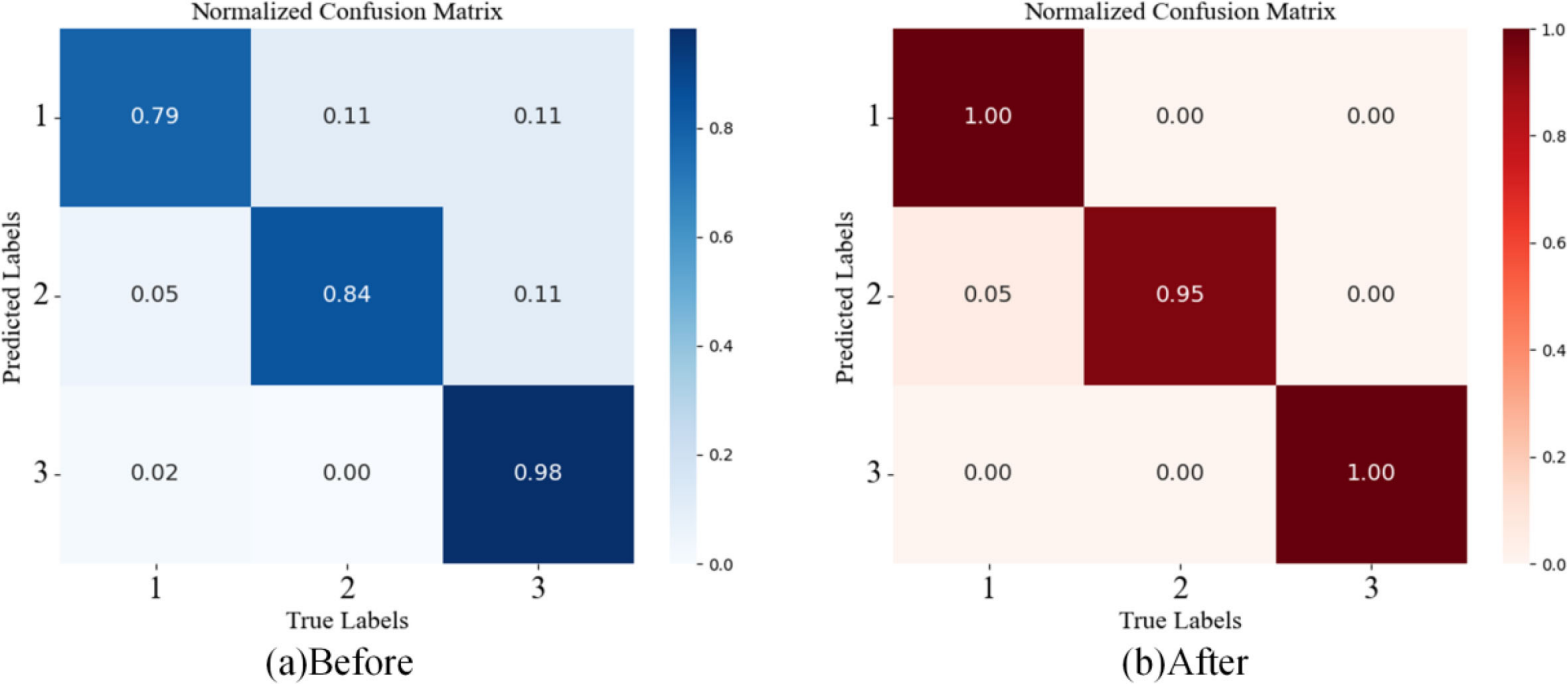
Confusion matrices before and after model improvement, **(a)** for ResNet34 and **(b)** for DH-GarlicNet.

By comparing the confusion matrices before and after model improvement, the changes in recognition performance become apparent. As shown in **Figure 7**, the original model’s confusion matrix exhibits an asymmetric structure, with an uneven distribution of intensity along the main diagonal and noticeable misclassification in the non-diagonal regions. Specifically, the model struggles with recognizing locally damaged garlic, highlighting its limitations in feature discrimination. The improved confusion matrix shows a more balanced structure, with generally enhanced diagonal elements and a more uniform intensity distribution, reflecting a comprehensive improvement in the model’s ability to recognize all categories. The misclassification pattern in the non-diagonal region has changed qualitatively, with systematic errors from the original model disappearing, leaving random misclassifications. This shift suggests that the improved model no longer has significant category bias or feature confusion. Visually, the original matrix has a more scattered color distribution, with significant variations in the main diagonal’s color gradient, while the improved matrix shows a clearer, more concentrated diagonal pattern with a more compact color distribution, indicating better feature space construction and clearer decision boundaries between categories.

**Figure 8:**
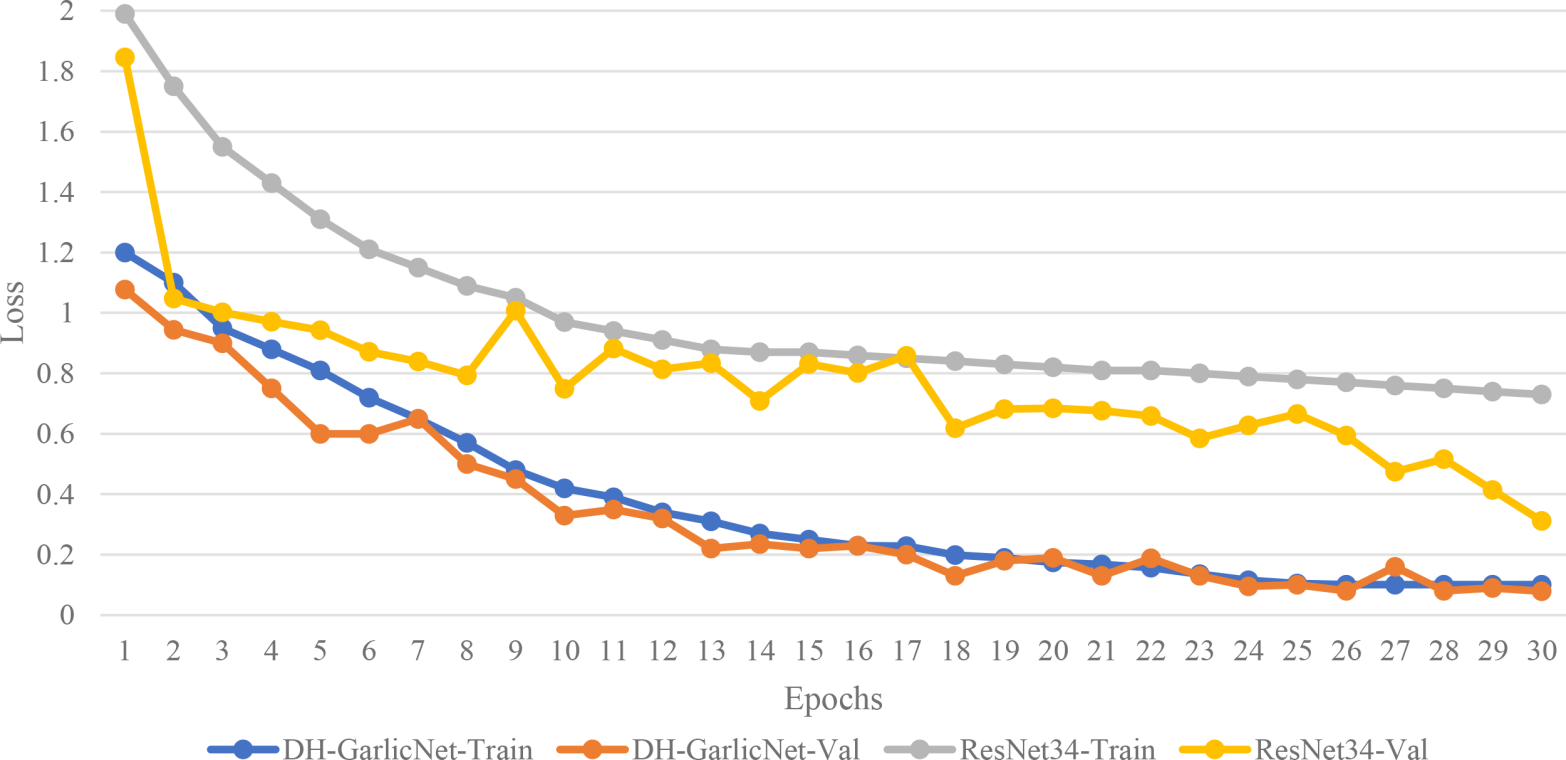
Loss curves before and after model improvement.

As shown in **Figure 8**, the loss curves are compared before and after the model improvement. The loss curve of the original model shows a typical fluctuating downward trend. In the early stage of model training, the decline speed is relatively fast, and then it presents a clear stable state in the later stage. Notably, in the later stages, the validation loss experiences intermittent rebounds, indicating instability in the model optimization. In contrast, the improved model shows a smoother and more stable decrease in loss. The loss drops more quickly in the early training stages, reflecting better parameter initialization. The curve maintains a consistent downward slope throughout the training, eventually converging to a lower final value, signaling enhanced generalization. The original model’s loss curve exhibits distinct “sawtooth” fluctuations, particularly in the middle stages, highlighting optimization instability. The improved curve, however, demonstrates a nearly monotonic descent with much smaller fluctuations, indicating that the improvements to the model have enhanced the optimization process, making training more stable and reliable. The main reason why the improved model did not suffer from overfitting lies in the fact that the loss curve during its training process exhibits excellent convergence characteristics and generalization ability. Specifically, the training loss and the validation loss remained close throughout the training process, and there was no significant bifurcation phenomenon between the two curves. This indicates that the model not only fits the training data but also maintains a low error rate for unseen validation data. Moreover, the validation loss did not show a rebound or continuous upward trend in the later training stage, but instead steadily decreased and converged to a lower level in sync with the training loss. This further confirms that the model did not overlearn the noise or details in the training set but learned effective features with generalization ability. In contrast, the validation loss of the original model showed intermittent rebounds in the later stage and had a large gap compared to the training loss, which is a typical manifestation of overfitting. Therefore, the improved model achieved a balance between training and validation performance through a more stable optimization process and more reasonable parameter initialization, thereby avoiding overfitting.

### 3.3 Comparative experiments with other algorithms

To systematically verify the performance of the DH-GarlicNet model proposed in this study in specific tasks and fully evaluate its technical advantages, we have carefully designed a rigorous comparative experimental program. Five representative classical convolutional neural networks are selected as benchmark models for comparative analysis, including AlexNet (Krizhevsky et al., 2017), RepVGGNet_A0 (Ding et al., 2021), MobileNetV4_s (Qin et al., 2024), FasterNet_T0 (Chen et al., 2023), and CoAtNet_0 (Dai et al., 2021). The experimental results show that compared to these mainstream models, our proposed DH- GarNet exhibits significant advantages in key performance metrics, not only achieving the highest recognition accuracy (up to 98.95%), but also maintaining the lowest loss value (only 0.0793), which fully validates the model’s sophistication and robustness for the damaged garlic feature extraction and classification tasks. Specific details are shown in **Table 7**.

**Table 7:** Comparison results with classical classification algorithms.

Model	Acc(%)	P(%)	R(%)	F1(%)	Loss
AlexNet	78.84	67.17	69.43	65.83	0.5207
RepVGGNet_A0	94.74	94.70	96.27	92.27	0.2673
MobileNetV4_s	75.79	50.00	58.17	53.67	0.5829
FasterNet_T0	87.37	84.57	78.53	80.70	0.3554
CoAtNet_0	97.89	96.50	96.30	96.20	0.1133
**DH-GarlicNet**	**98.95**	**98.23**	**98.03**	**98.10**	**0.0793**

The bolded parts indicate the recognition results of the DH-GarlicNet model proposed in this study.

To deeply analyze the performance of each model in the garlic classification task, this study calculated the classification metrics, including precision, recall, and F1 score, of six comparison models (including DH-GarlicNet and five benchmark models) on three key categories (localized damage, root damage, and normal garlic), and the detailed data are shown in **Table 8**. The quantitative analysis results show that our proposed DH-GarlicNet model demonstrates comprehensive advantages: in terms of precision metrics, DH-GarlicNet achieves the highest values in two of the three categories (localized damage and intact), both reaching 1.000; in terms of recall, the model achieves the highest values in two of the three categories (root damage and intact), both reaching 1.000; the F1 score, a composite metric, also shows that DH-GarlicNet maintains the lead in all three categories, with intact garlic reaching 1.000. In contrast, the other benchmark models show varying degrees of limitations, e.g., MobileNetV4_s achieves a high recall in the category of intact garlic, but the overall recognition performance is poor. These data fully validate the innovation of the DH-GarlicNet network architecture, which effectively improves the model’s ability to discriminate various types of damage features through the feature fusion module and the multiscale module, and realizes a comprehensive and balanced classification performance improvement.

**Table 8:** Comparison results with the classical model for each category.

Model	Index	Local damage	Root damage	Whole
RepVGGNet_A0	P	1.000	0.889	0.952
	R	0.824	0.889	1.000
	F1	0.904	0.889	0.975
AlexNet	P	0.500	0.533	0.982
	R	0.294	0.889	0.900
	F1	0.370	0.666	0.939
MobileNetV4_s	P	0.000	0.737	0.763
	R	0.000	0.778	0.967
	F1	0.000	0.757	0.853
FasterNet_T0	P	0.706	0.923	0.908
	R	0.706	0.667	0.983
	F1	0.706	0.774	0.944
CoAtNet_0	P	0.895	1.000	1.000
	R	1.000	0.889	1.000
	F1	0.945	0.941	1.000
DH-GarlicNet	P	1.000	0.947	1.000
	R	0.941	1.000	1.000
	F1	0.970	0.973	1.000

### 3.4 Comparative experiments on activation functions

In this study, the mechanism of the influence of different nonlinear transformations on the performance of the garlic image recognition model is deeply investigated by constructing a comprehensive activation function evaluation system. Under the experimental conditions of strictly controlling variables, we keep all hyperparameters of the DH-GarlicNet network architecture unchanged, and only replace the SiLU activation function with nine representative activation functions in turn, including: classical segmented linear functions (ReLU, ReLU6), improved ones with negative intervals (ELU, LeakyReLU), continuous smooth functions proposed in recent years (Mish, GELU), traditional S-shaped functions (Sigmoid), and hardware-optimized functions (Hardswish, Hardsigmoid). This systematic comparison scheme covers the current mainstream activation function types and ensures the broad applicability of the research findings. Specific details are shown in **Table 9**.

**Table 9:** Comparison results with classical activation functions.

Activation function	Acc(%)	P(%)	R(%)	F1(%)	Loss
ELU	96.84	96.10	95.50	95.57	0.1559
ReLU	97.89	96.17	96.17	96.17	0.1453
LeakyReLU	95.79	95.60	92.17	93.27	0.1779
Mish	95.79	93.97	95.07	94.50	0.1245
GELU	96.84	95.77	94.33	94.90	0.1174
ReLU6	97.89	97.70	96.07	96.73	0.1191
Sigmoid	69.47	41.33	48.33	44.40	0.7597
Hardsigmoid	63.16	21.07	33.33	25.83	0.9247
Hardswish	96.84	95.00	97.03	95.77	0.1619
**SiLU(DH-GarlicNet)**	**98.95**	**98.23**	**98.03**	**98.10**	**0.0793**

The bolded parts indicate the recognition results of the DH-GarlicNet model proposed in this study.

The experimental data show that the SiLU model achieves an average accuracy of 98.95%, which is significantly better than other activation functions (Mish: 95.79%, GELU: 96.84%). It is worth noting that the different activation functions show a clear performance gradient between them. The worst performer, Hardsigmoid, only achieves 63.16% recognition accuracy with a validation loss of 0.9247, which is mainly attributed to three defects: firstly, the hard saturation property leads to more than 38% of neurons falling into a “dead” state during the training process; secondly, the fixed output range (0,1) severely restricts the ability of feature characterization; and lastly, the non-smooth transition point makes the gradient propagation accuracy decrease. In contrast, although Mish and GELU, which have the second-best performance, also possess continuous smooth characteristics, there is an obvious gap in feature selection capability due to the lack of an adaptive gating mechanism of SiLU.

The experimental results show that for the garlic image analysis task that needs to deal with continuous gradient features, an activation function with the following characteristics at the same time can achieve the best results: (1) smooth gradient transfer; (2) adaptive feature scaling; and (3) moderate nonlinear transformation.

### 3.5 Comparative experiments on attentional mechanisms

To systematically assess the impact of different attention mechanisms on garlic recognition performance, this study performed a series of controlled experiments within the DH-GarlicNet framework. By keeping all other network components consistent, we replaced the SE module with five distinct attention mechanisms: CA, ECA, SAM, GAM, and SimAM. Detailed information is provided in **Table 10**. The results reveal that the SE module outperforms all the other models. A notable performance gap was observed between the various attention mechanisms, with GAM demonstrating the weakest performance—achieving only 58.95% recognition accuracy and a validation loss of 0.9137. GAM’s limited performance is likely due to overfitting, stemming from the increased number of parameters introduced by its complex spatial-channel dual-attention mechanism. Additionally, the spatial-attention module overly emphasizes local features, neglecting the global context essential for garlic recognition. Furthermore, the multilayer perceptron design adds unnecessary computational complexity during feature interaction. In contrast, the SE module offers a better balance between computational efficiency and feature selection by using a lightweight channel attention design. These experiments not only confirm the SE module’s suitability for garlic image recognition but also highlight the relationship between the design principles of different attention mechanisms and the task at hand. The findings suggest that channel attention mechanisms are more effective for garlic damage recognition tasks, where balancing global features and local details is critical.

**Table 10:** Comparative results with classical attention mechanisms.

Attention mechanism	Acc(%)	P(%)	R(%)	F1(%)	Loss
CA	97.89	97.60	96.17	96.87	0.1581
ECA	96.84	95.00	94.43	94.27	0.1453
SAM	95.79	95.00	92.37	93.57	0.1941
GAM	58.95	30.80	35.30	31.77	0.9137
SimAM	95.79	95.00	92.37	93.57	0.1283
**SE(DH-GarlicNet)**	**98.95**	**98.23**	**98.03**	**98.10**	**0.0793**

The bolded parts indicate the recognition results of the DH-GarlicNet model proposed in this study.

### 3.6 Recognition effect demonstration

**Figure 9:**
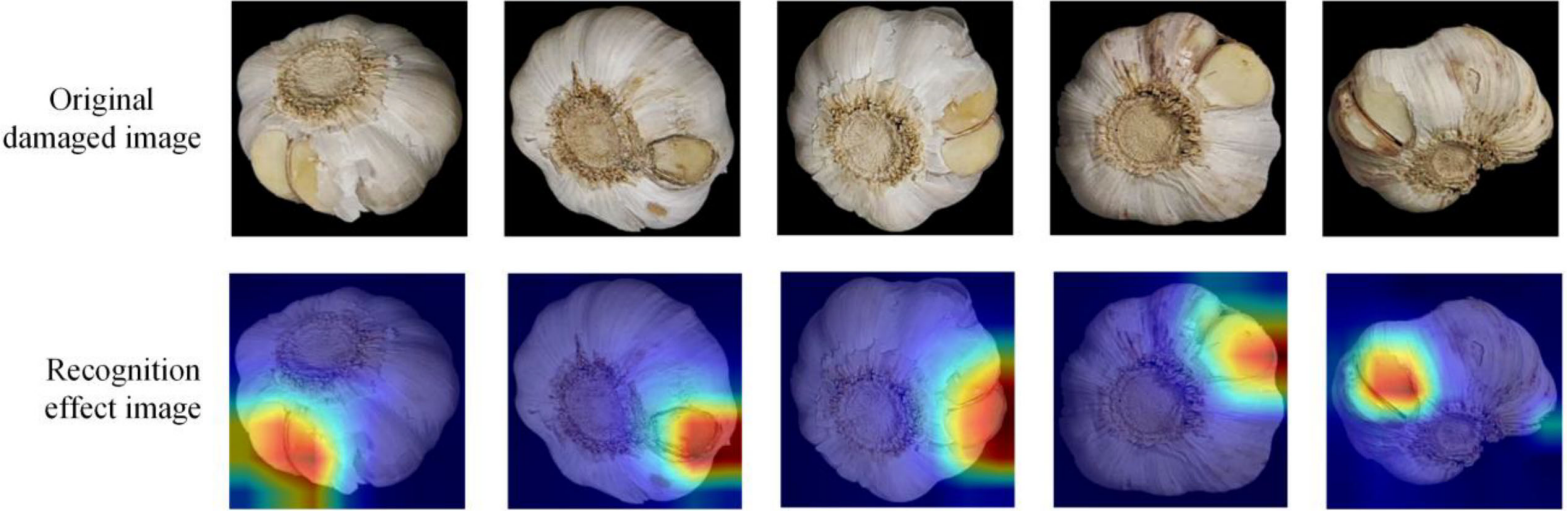
Demonstration of the damage area recognition effect.

To investigate the effectiveness of the model improvement for garlic damage recognition, this study utilized the gradient-weighted class activation mapping (Grad-CAM) technique for a systematic visualization and analysis (Wang and Zhang, 2023). Grad-CAM, an advanced deep learning visualization method, computes the gradient information of the target class relative to the feature map of the final convolutional layer. These gradients are then used as weights to combine the feature maps, producing an intuitive heat map. This technique not only highlights the critical regions of the image that influence the model’s decision but also shows the extent of the network’s contribution to the prediction. As shown in **Figure 9**, the enhanced model demonstrates notable improvements in damage localization. The heat map indicates that DH-GarlicNet can accurately target the garlic damage area, with its region of interest aligning closely with the actual damage site. Notably, the model is highly sensitive to detecting small damages, which is primarily due to its enhanced deep feature extraction capability. The gradient distribution, ranging from blue (low attention) to red (high attention) in the heat map, illustrates the model’s ability to assess varying damage levels quantitatively.

## 4 Discussion

Although DH-GarlicNet demonstrates promising performance in damaged garlic recognition, several limitations in dataset construction and algorithm design warrant further discussion.

Regarding dataset limitations, the current garlic image database may lack sufficient coverage of real-world scenarios. The samples were primarily collected under controlled lighting conditions, failing to adequately represent the complex and variable imaging conditions encountered in actual agricultural production environments, such as uneven natural illumination, shadow interference, and multi-angle field photography. Furthermore, the defined damage categories in the dataset may oversimplify the complex spectrum of garlic defects observed in practice. For instance, subtle distinctions between early-stage mold contamination and surface mechanical damage may not be sufficiently characterized. The absence of temporal sequence data documenting damage progression also constrains the model’s ability to identify developing defects, which is particularly crucial for early-stage damage warning.

In terms of algorithmic design, future research should focus on lightweight improvements to DH-GarlicNet to enhance deployment efficiency in practical agricultural applications. For lightweight design, depthwise separable convolutions could replace conventional convolution operations, significantly reducing parameters by decoupling spatial and channel correlations. Combined with channel pruning techniques, redundant channels could be dynamically trimmed based on attention weights, maintaining accuracy while reducing computational complexity.

Regarding network architecture, a lightweight backbone based on inverted residual structures could be developed, employing linear bottleneck layers and dilated convolutions to balance feature representation capability with computational efficiency. An adaptive receptive field mechanism could be introduced, enabling dynamic adjustment of convolutional kernel ranges according to damage region distribution in input images, thereby avoiding computational waste from fixed-scale convolutions.

For attention module optimization, a lightweight hybrid attention mechanism could be designed by decoupling channel and spatial attention, with computation overhead reduced through grouped convolutions and parameter sharing strategies. Specifically, a local-global alternating attention mechanism could be developed for garlic damage characteristics, computing fine-grained attention only in key regions rather than across the entire image.

In model compression, knowledge distillation techniques could be employed, using the original large model as a teacher network to guide the training of lightweight student networks. By designing specialized distillation loss functions for damage features, student networks could better inherit the teacher network’s discriminative capability for subtle defects. Additionally, quantization-aware training could help models adapt to low-bit deployment environments, significantly improving inference speed while maintaining recognition accuracy.

These lightweight improvements would make the model more suitable for deployment on farmland edge computing devices or mobile inspection terminals, enabling real-time online detection of garlic damage and providing more efficient technical support for smart agriculture applications. Future research could also explore the integration of lightweight models with multimodal data (e.g., near-infrared, hyperspectral) to further enhance detection robustness in complex scenarios.

## 5 Conclusions

The DH-GarlicNet model proposed in this research demonstrates outstanding performance in garlic damage detection. By integrating DWConv, SE module, and SiLU activation function, it achieves a detection accuracy of 98.95%, surpassing traditional algorithms and showing significant performance improvement. To further assess the model’s effectiveness, we conducted ablation and extensive comparison experiments to analyze the contribution of each module. The results reveal a clear synergistic effect among the components, with the deep convolutional structure enhancing feature extraction, the SE module improving focus on critical areas, and the SiLU activation function refining the model’s ability to represent nonlinear features. These improvements collectively lead to a substantial increase in detection accuracy. Moreover, DH-GarlicNet is evaluated using a confusion matrix and loss curve, showing excellent recognition and fitting performance. Grad-CAM visualization further confirms the model’s ability to precisely focus on and highlight the damage regions, validating its proficiency in local feature learning and target recognition. Overall, these findings suggest that DH-GarlicNet excels in garlic damage detection and has promising application potential.

## Data Availability

The original contributions presented in the study are included in the article/supplementary material. Further inquiries can be directed to the corresponding author.
